# Pantoprazole before Endoscopy in Patients with Gastroduodenal Ulcer Bleeding: Does the duration of Infusion and Ulcer Location Influence the Effects?

**DOI:** 10.1155/2012/561207

**Published:** 2012-10-17

**Authors:** Istvan Rácz, Milan Szalai, Nora Dancs, Tibor Kárász, Andrea Szabó, Mihaly Csöndes, Zoltan Horváth

**Affiliations:** ^1^Department of Gastroenterology, Petz Aladár Teaching Hospital, Győr 9024, Hungary; ^2^Department of Mathematics and Computer Sciences, Széchenyi István University, Győr 9026, Hungary

## Abstract

The aim of this study was to investigate the effect of preemptive pantoprazole infusion on early endoscopic findings in patients with acute ulcer bleeding. Records of 333 patients admitted with acute ulcer bleeding were analyzed. Ulcer bleeders were given either 80 mg bolus of pantoprazole followed by continuous infusion of 8 mg per hour or saline infusion until endoscopy. In 93 patients saline infusion whereas in 240 patients bolus plus infusion of pantoprazole was administrated with mean (±SD) durations of 5.45 ± 12.9 hours and 6.9 ± 13.2 hours, respectively (*P* = 0.29). Actively bleeding ulcers were detected in 46/240 (19.2%) of cases in the pantoprazole group as compared with 23/93 (24.7%) in the saline infusion group (*P* = 0.26). Different durations of pantoprazole infusion (0–4 hours, >4 hours, and >6 hours) had no significant effect on endoscopic and clinical outcome parameters in duodenal ulcer bleeders. Gastric ulcer bleeders on pantoprazole infusion longer than 4 and 6 hours before endoscopy had actively bleeding ulcers in 4.3% and 5% compared to the 19.5% active bleeding rate in the saline group (*P* = 0.02 and *P* = 0.04). Preemptive infusion of high-dose pantoprazole longer than 4 hours before endoscopy decreased the ratio of active bleeding only in gastric but not in duodenal ulcer patients.

## 1. Introduction

In patients with bleeding peptic ulcers infusion of high-dose proton pump inhibitor (PPI) after endoscopic hemostasis reduces recurrent bleeding and improves clinical outcomes [1–6]. High-dose proton pump inhibitors administered intravenously increase and maintain gastric pH above 6, which is thought to be beneficial for platelet aggregation and clot formation over bleeding vessels [7–9]. Recent studies have shown that not only postendoscopic but also preendoscopic high-dose proton pump inhibitor therapy significantly reduces the proportion of patients with high-risk stigmata of recent hemorrhage (active bleeding, nonbleeding visible vessel, and adherent clot) at early endoscopy and decreases the need for endoscopic intervention [10, 11]. However, almost no data exist about the minimally required duration of PPI infusion before endoscopy, whose results downstage the endoscopic lesions and decrease the need for endoscopic intervention [12]. We hypothesized that patients undergoing endoscopy almost immediately after PPI administration may likely utilize only few benefits whereas longer duration of preendoscopic PPI infusion more likely generates clinical benefits.

The aim of our retrospective case control study was to investigate the effects of preemptive infusion of pantoprazole before endoscopy on early endoscopic findings and clinical outcomes in patients with gastroduodenal ulcer bleeding. We also aimed to estimate the threshold duration of pantoprazole infusion before endoscopy ensuring downstage of endoscopic lesions. Furthermore, we separately analyzed the effect of preendoscopic pantoprazole infusion in patients with gastric and duodenal ulcer bleeding.

## 2. Patients and Methods

### 2.1. Study Design

This was a single-center, retrospective, comparative cohort study. The study protocol was approved by the Regional Ethics Committee of the Petz Aladár Teaching Hospital. There was no pharmaceutical industry support for this study.

### 2.2. Patients

Data collection was carried out by use of the files of those patients who presented with the signs of acute upper gastrointestinal bleeding (i.e., melena or hematemesis with or without hypotension) during the evening and night hours (6 PM–8 AM) at the Gastroenterology Emergency Department of the Petz Aladár Teaching Hospital in Győr. All patients were evaluated by admitting as residents according to the actual patient managing protocols. Patients with hypotensive shock (systolic RR ≤90 Hgmm or pulse ≥110 beats per minute) were initially resuscitated to stabilize their condition. Patients with continuous shock symptoms despite resuscitation activity underwent immediate urgent endoscopy by the on-call endoscopy team and these patients were excluded from the study analysis.

According to the hospital practice protocol for patients who had bleeding ulcers associated with intake of nonsteroidal anti-inflammatory drugs (NSAIDs) or aspirin, the drugs were discontinued. Fresh-frozen plasma was given to those bleeders who were on coumarin therapy. Anticoagulation-dependent cardiac or postthrombosis patients underwent low-molecular-weight heparin therapy until the bleeding was stabilized.

Medical records of 1369 consecutive patients admitted with upper gastrointestinal bleeding between April 2007 and July 2011 were retrospectively analyzed. In this time period all diagnostic and therapeutic procedures were performed by the same hospital managing protocol except the use of intravenous proton pump inhibitor (PPI) while awaiting an early endoscopy. The preemptive PPI use was included in the managing protocol of the gastrointestinal bleeders arriving during the evening and night time in January 2009. Since that time all acute bleeders with the suspicion of upper GI bleeding were given an 80 mg intravenous bolus injection of pantoprazole followed by continuous infusion of 8 mg per hour until endoscopic examination the next morning. Before that, during 2007 and 2008 saline infusion was administrated to all patients during the preendoscopy hospital period and PPI infusion was initiated only after endoscopy in patients with ulcer bleeding. This difference in the preendoscopy infusion policy offers the possibility to evaluate whether high-dose intravenous pantoprazole before endoscopy would have a therapeutic effect on bleeding ulcers, reduce the need for endoscopic therapy, and result in improved clinical outcomes. 

Use of pantoprazole or saline was started at admission and was continuously given also during the endoscopy. In patients with signs of ongoing bleeding (i.e., repeated hematemesis or fresh blood in the nasogastric tube) an urgent endoscopy was performed by the endoscopic on-call team. In all other cases with stabilized condition the early endoscopy was performed next morning by expert endoscopists.

At endoscopy the Forrest classification was used to define the bleeding ulcers. Those ulcers with spurting bleeding, oozing bleeding, or nonbleeding visible vessels (Forrest Ia, Ib, and IIa) were injected with 1 : 10.000 diluted epinephrine followed by coaptive thermocoagulation or hemoclip application.

Hemostasis was considered successful if bleeding had stopped and if the visible bleeding vessels disappeared or were cavitated. Clots covering ulcers were firmly irrigated by water after injection with diluted epinephrine around the ulcer and underlying vessels, if the present were treated. To examine whether *Helicobacter pylori* (*H. pylori*) infection was present antral biopsies were taken for rapid urea test and histology. 

Patients with no need for endoscopic therapy were transferred to general medical wards. Those patients who underwent endoscopic hemostasis were admitted to the high-dependency gastroenterology ward for monitoring. For those patients who required endoscopic ulcer hemostasis 8 mg per hour pantoprazole was infused for 72 hours after endoscopy.

Rebleeding was considered if any of the following events occurred: repeated vomiting of fresh blood, hypotensive shock (defined as systolic blood pressure ≤90 Hgmm or a pulse ≥110 beats per minute) with melena after stabilization, or decrease in the hemoglobin level of more than 2 g/dL within 24 hours after transfusion, resulting in a hemoglobin level of 10 g/dL or less. Patients who were judged to be rebleeders underwent repeated endoscopy either by the on-call duty team or by the regular daily expert endoscopists. Rebleeding was confirmed in cases with Forrest Ia, Ib ulcers, or if there was fresh blood in the stomach either with a Forrest IIa or IIb ulcer. Endoscopic hemostatic therapy was repeated in cases with rebleeding ulcers. Surgery was indicated if the bleeding could not be controlled endoscopically or in cases with second rebleeding.

After the 72-hour infusion of pantoprazole, patients were given 40 mg per day pantoprazole orally for 8 weeks. The same oral therapy was administrated in cases with Forrest IIc and III ulcers at first endoscopy. In patients who were *H. pylori* positive according to either the rapid urease test or by histology a one-week eradication therapy (2 × 40 mg pantoprazole twice daily and 500 mg clarithromycin and 1 g amoxicillin twice daily) was started 3 days after the onset of bleeding. That was followed by 40 mg/day-pantoprazole treatment for the remaining six weeks. Patients were regularly followed up to 30 days after hospital admission by the contact of the family doctors or by their controls in the hospital outpatient offices. Clinic followup, hospital readmissions, or death were checked and verified through the computerized hospital record system.

In our retrospective analysis the primary outcome was the active bleeding at the first endoscopic examination. Secondary outcomes included the need for endoscopic hemostasis at the first endoscopy, need for urgent endoscopy, rates of rebleeding, need for emergency surgery, and death from any reason within 30 days of hospital admission. Both primary and secondary outcomes were analyzed also in subgroups of patients with different durations of intravenous pantoprazole administration before the first endoscopy. Furthermore, all these analyses were performed separately both in duodenal and gastric ulcer patients.

### 2.3. Statistical Analysis

Student's *t* test was used to analyze age, hemoglobin, and shock symptoms. All other parameters were primarily analyzed with Chi-square test and the latter was replaced with Fischer's exact test when the numbers of data were insufficient. All tests of significance were two tailed, and a *P* value of 0.05 was considered to indicate statistical significance.

## 3. Results

**Figure 1:**
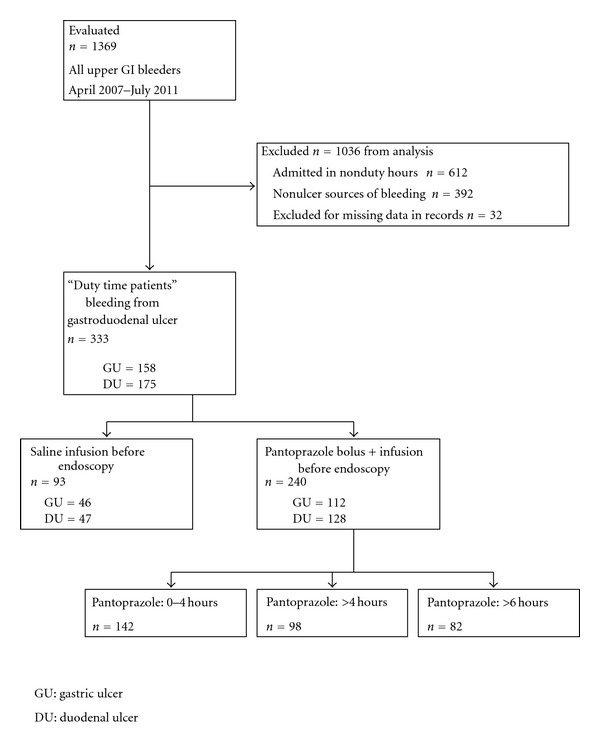
Patient distribution and groups of retrospective analysis.

Between April 2007 and July 2011 a total of 1369 patients were admitted to our emergency unit with the signs of acute upper gastrointestinal bleeding. A total of 1036 patients were excluded from our retrospective analysis: a total of 612 patients were admitted in nonduty hours; in 392 patients the endoscopy detected nonulcer sources of bleeding, and in 32 cases essential data were missing in their records. Finally, clinical and endoscopic data were analyzed for those 333 gastroduodenal ulcer bleeding patients who were admitted in duty hours and the endoscopy examinations were done either in the next morning or urgently. Before endoscopy saline infusion was administrated in 93 patients whereas in 240 patients bolus plus infusion pantoprazole was started (Figure 1).

Demographic and clinical characteristics were similar in the two groups (Table 1). The source of bleeding was duodenal ulcer in 47 of 93 patients (50.5%) in the saline infusion group and 128 of 240 (53.3%) in the pantoprazole group. The mean (±SD) duration of infusion before endoscopy was 5.45 ± 12.9 hours in the saline group and 6.9 ± 13.2 hours in the pantoprazole group (*P* = 0.29).

Among the 240 patients in the pantoprazole group during the first endoscopic examinations actively bleeding ulcers were detected in 46 (19.2%) cases, as compared with 23 of the 93 patients (24.7%) in the saline infusion group (*P* = 0.26). The ratio of ulcers with nonbleeding visible vessels, clots, pigmented spots, and clean base also did not differ significantly in the two groups (Table 2). At the first endoscopy 147 of 240 patients (61.3%) in the pantoprazole group and 53 of 93 patients (56.9%) in the saline group required endoscopic treatment (*P* = 0.82). Urgent endoscopy was performed in 19 (7.9%) pantoprazole group patients and in 8 (8.6%) in the saline group patients (*P* = 0.89).

Emergency surgery was performed in 21 patients (8.8%) in the pantoprazole and in 10 patients (10.7%) in the saline group (*P* = 0.57). Recurrent bleeding occurred in 40 patients (16.7%) in the pantoprazole group and 13 patients (13.9%) in the saline group (*P* = 0.55). Within 30 days after hospital admission 15 patients (6.3%) in the pantoprazole group and 4 patients (4.3%) in the saline group died (*P* = 0.49). 

When analyzing all ulcer patients (*n* = 333) the duration of pantoprazole infusion had no significant effect on outcome parameters compared to the saline infusion patients except the significantly increased ratio of ulcers with clot in patients who were on pantoprazole infusion up to 4 hours (25.3% versus 12.9%; *P* = 0.02).

In the subgroup of the 175 duodenal ulcer patients the preemptive bolus + pantoprazole infusion therapy compared to saline infusion had no significant effect neither on endoscopic appearance of ulcers nor on clinical outcome measures. In duodenal ulcer bleeders the duration of pantoprazole infusion (0–4 hrs, >4 hrs, and >6 hrs) also had no significant effect on the endoscopic appearance of ulcers at the first endoscopy and on clinical outcomes (Table 3).

In contrary, when analyzing the subgroup of the 158 gastric ulcer bleeders in those patients who were on pantoprazole infusion longer than 4 and 6 hours the ratios of actively bleeding ulcers were only 4.3% and 5% compared to the 19.5% actively bleeding ulcer rate in the saline infusion group (*P* = 0.02 and *P* = 0.04) (Table 4). In addition, a significantly higher proportions of gastric ulcers were covered by clot at the first endoscopy in those patients who received pantoprazole infusion up to 4 hours compared to those gastric ulcer bleeders on saline infusion (30.8% versus 10.9%, *P* = 0.01). Similarly to duodenal ulcer bleeders in patients with gastric ulcer bleeding any analyzed duration of the preendoscopic pantoprazole infusion had no significant modification on clinical outcomes as observed with saline infusion only.

## 4. Discussion

Our retrospective analyses only partly reaffirm the efficacy of parenteral PPI treatment initiated before the endoscopy for gastroduodenal ulcer bleeding. In our study early administration of high-dose pantoprazole did not reduce significantly the ratio of active bleeding and the need for endotherapy at the first endoscopy among the total cohort of gastroduodenal ulcer bleeders compared to the saline infusion group. Moreover, no significant differences were detected between the two patient cohorts in any clinical outcomes.

In a placebo-controlled randomized clinical trial that involved endoscopic therapy, Lau et al. found that fewer cases of actively bleeding peptic ulcers were seen among patients who received high-dose i.v. omeprazole before endoscopy than among those who had received placebo [10]. In this study early administration of high-dose omeprazole also reduced the need for endoscopic therapy but had no beneficial effect on clinical outcomes. A recent Cochrane meta-analysis of six randomized trials of preendoscopic PPI therapy also found the same results [11]. 

The notion that acid suppression facilitates clot formation and confers clot stability over arteries in bleeding peptic ulcers is the theoretical background of i.v. PPI therapy either in postendoscopic and preendoscopic settings [7–9, 13]. 

Regarding preemptive i.v. PPI treatment one key variable for which there exist only limited data is the time elapsed on PPI therapy until endoscopy [12]. In the Lau trial the mean duration of intravenous omeprazole administration before the endoscopy was 14.7 ± 6.3 hours which is more than twice as long as the mean duration of i.v. pantoprazole infusion therapy in our study (6.9 ± 13.2 hours). It can be postulated that if patients undergo endoscopy within only few hours or almost immediately after PPI administration itmay likely utilize only few clinical benefits. Alternatively, if patients undergo endoscopy more later after the start of PPI treatment the more clinical benefits can be achieved. In our retrospective analysis we tried to find the threshold duration of pantoprazole administration being effective either in primary or in secondary outcomes. Pantoprazole infusion durations (0–4 hours, >4 hours, and >6 hours) had no significant downstaging effect on endoscopic lesions except increased early clot formation ratio when compared to saline infusion. However, a tendency of less active bleeding was seen with longer pantoprazole treatment in the total cohort of gastroduodenal bleeders. 

The differences between the Lau study and our results regarding the proportion of actively bleeding ulcers and the need for endoscopic therapy at first endoscopy can be explained at least partly by the markedly different durations of preemptive PPI therapy. 

Longer duration of PPI treatment in the Lau study resulted in significant benefits. Analyzing and comparing the results of both studies one may speculate that the threshold duration of effective preendoscopic PPI treatment may therefore exist in between 6–14 hours.

The initial step of hemostasis is clot formation on ulcers in pH dependent. Data from in vitro studies have shown that gastric pH above 6 is the optimal for platelet aggregation [7–9]. Avgerinos et al. performed a clinical trial to examine the rapidity and maintain intragastric pH elevation in gastroduodenal ulcer bleeding patients receiving an initial 80 mg bolus injection followed by 8 mg/h continuous infusion of pantoprazole [14]. In their study the mean percentage of time spent above pH 6.0 during the first 12 hours of the pantoprazole infusion period was only 43.3%. For the time period of 0–4 hours the mean pH in the fundus was not higher than in the placebo group. These results suggest that the hemostatic effect of preemptive parenteral PPI therapy might be not only pH dependent but also time dependent.

According to our knowledge our study is the first which separately analyzed the effects of preendoscopic pantoprazole infusion in the subgroups of duodenal and gastric ulcer bleeders. Similarly to the total cohort of all gastroduodenal bleeders independently of pantoprazole infusion duration no significant modification of the outcome measures was seen in the 128 patients bleeding from duodenal ulcers compared to the saline group. In contrary, longer than 4 hours duration of pantoprazole infusions significantly reduced the ratio of active bleeding at first endoscopy among those 112 patients who bled from gastric ulcers. These latter results may reflect that clot formation activity related to pantoprazole-induced acid suppression has different rapidity and potential in the stomach compared to the duodenum.

Several factors limit the value of our findings. First, it was a retrospective study with typical inherent limitations of the retrospective analysis. Second, long-term aspirin and NSAID as well as anticoagulant users were not excluded from the analysis; therefore patients enrolled in our study may have been at different risks of ulcer bleeding. The effect of high-dose pantoprazole on clot formation and stability in patients taking aspirin or NSAIDs is unknown.

Third, we only analyzed the data of those ulcer bleeding patients who were admitted on duty hours. However, one advantage of our study is that it reflects the real life situation using high-dose PPI inhibitors as a replacement of immediate urgent endoscopy during the night hours.

In conclusion, according to our retrospective analysis profound acid suppression in gastroduodenal ulcer bleeding patients awaiting endoscopy did not decrease significantly the ratio of active bleeding and the need for endoscopic therapy; however a trend of less active bleeding was seen with longer pantoprazole treatment. Preemptive administration of high-dose pantoprazole for longer than 4 hours decreased the severity of bleeding at first endoscopy in gastric ulcer patients but not in duodenal ulcer patients.

## Figures and Tables

**Table 1:** Characteristics of 333 gastroduodenal ulcer bleeding patients included in the retrospective analysis.

Characteristic	Pantoprazole (*n* = 240)	Saline (*n* = 93)	*P* value
Age—year	63.4 ± 15.2	66.0 ± 13.4	0.12

Male sex—number (%)	175 (72.9%)	59 (63.4%)	0.08

Hemoglobin—g/dL	95.3 ± 30.1	96.8 ± 30.4	0.68

Shock symptoms—number of patient (%)	22 (9.2%)	3 (3.2%)	0.06

Coexisting illness—number of patients (%)			
Cirrhosis	40 (16.6%)	16 (17.2%)	0.90
Cardiovascular	146 (60.8%)	58 (62.3%)	0.79

*Helicobacter pylori* infection—number of patients (%)	73 (30.4%)	33 (35.4%)	0.37

Risk factors for bleeding—number of patients (%)			
NSAID and/or aspirin	120 (50.0%)	42 (45.2%)	0.42
Anticoagulant	19 (7.9%)	8 (8.6%)	0.83
Previous gastroduodenal ulcer	74 (30.8%)	28 (30.1%)	0.89

Source of bleeding			
Duodenal ulcer	128 (53.3%)	47 (50.5%)	0.64
Gastric ulcer	112 (46.7%)	46 (49.5%)	0.64

Symptom at presentation—number of patients (%)			
Melena	177 (73.7%)	65 (69.9%)	0.47
Hematemesis	111 (46.2%)	36 (38.7%)	0.21
Both	60 (25.0%)	16 (17.2%)	0.12

Duration of infusion before endoscopy (hours)	6.9 ± 13.2	5.45 ± 12.9	0.29

**Table 2:** Outcomes with different durations of pantoprazole infusion compared to saline infusion for the total of 333 gastroduodenal ulcer bleeders.

Outcome (all ulcer bleeders)	Saline	Pantoprazole all		Pantoprazole 0–4 hrs		Pantoprazole >4 hrs		Pantoprazole >6 hrs	
	(*n* = 93)	(*n* = 240)	*P* value	(*n* = 142)	*P* value	(*n* = 98)	*P* value	(*n* = 82)	*P* value
	Number of pts. (%)			Number of pts. (%)		Number of pts. (%)		Number of pts. (%)	
Endoscopic signs of bleeding									
Active bleeding	23 (24.7%)	46 (19.2%)	0.26	32 (22.5%)	0.69	14 (14.3%)	0.07	12 (14.6%)	0.09
Nonbleeding visible vessel	18 (19.3%)	49 (20.4%)	0.83	29 (20.4%)	0.84	20 (20.4%)	0.86	19 (23.2%)	0.54
Clot	12 (12.9%)	52 (21.7%)	0.50	36 (25.3%)	**0.02**	16 (16.3%)	0.50	14 (27.1%)	0.44
Pigmented spot and clean base	40 (43.0%)	93 (38.8%)	0.48	45 (31.6%)	0.08	48 (48.9%)	0.41	37 (45.1%)	0.78
Urgent endoscopy	8 (8.6%)	19 (7.9%)	0.89	12 (8.4%)	0.91	7 (7.1%)	0.84	5 (6.1%)	0.64
Recurrent bleeding	13 (13.9%)	40 (16.7%)	0.55	21 (14.8%)	0.86	19 (19.4%)	0.32	17 (20.1%)	0.24
Emergency surgery	10 (10.7%)	21 (8.8%)	0.57	13 (9.2%)	0.68	8 (8.2%)	0.54	7 (8.5%)	0.62
Mortality	4 (4.3%)	15 (6.3%)	0.49	8 (5.6%)	0.65	7 (7.1%)	0.39	5 (6.1%)	0.59

*P* values were calculated for comparison between saline infusion group and pantoprazole infusion group patients with different durations of pantoprazole administration.

**Table 3:** Outcomes for the 175 duodenal ulcer bleeders with different durations of pantoprazole infusion compared to saline infusion.

Outcome (duodenal ulcer bleeders)	Saline	Pantoprazole all		Pantoprazole 0–4 hrs		Pantoprazole >4 hrs		Pantoprazole >6 hrs	
	(*n* = 47)	(*n* = 128)	*P* value	(*n* = 77)	*P* value	(*n* = 51)	*P* value	(*n* = 42)	*P* value
	Number of pts. (%)			Number of pts. (%)		Number of pts. (%)		Number of pts. (%)	
Endoscopic signs of bleeding from duodenal ulcers									
Active bleeding	14 (29.8%)	31 (24.2%)	0.46	19 (24.6%)	0.53	12 (23.5%)	0.48	10 (23.8%)	0.53
Nonbleeding visible vessel	7 (14.8%)	27 (21.1%)	0.36	17 (22.0%)	0.33	10 (19.6%)	0.54	9 (21.4%)	0.42
Clot	7 (14.8%)	24 (18.7%)	0.55	16 (20.8%)	0.41	8 (15.7%)	0.91	8 (19.0%)	0.60
Pigmented spot and clean base	19 (40.4%)	46 (35.9%)	0.59	25 (32.5%)	0.36	21 (0.41%)	0.94	15 (35.7%)	0.65
Urgent endoscopy	5 (10.6%)	11 (8.5%)	0.64	6 (7.8%)	0.52	5 (9.8%)	0.82	3 (7.1%)	0.54
Recurrent bleeding	9 (19.1%)	22 (17.2%)	0.76	11 (14.2%)	0.47	11 (21.5%)	0.77	10 (23.8%)	0.59
Emergency surgery	8 (17.0%)	15 (12.3%)	0.36	8 (10.4%)	0.28	7 (13.7%)	0.81	6 (14.3%)	0.72
Mortality	3 (6.3%)	7 (5.7%)	0.76	5 (6.4%)	0.98	2 (3.9%)	0.58	1 (2.3%)	0.36

*P* values were calculated for comparison between saline infusion group and pantoprazole infusion group patients with different durations of pantoprazole administration.

**Table 4:** Outcomes for the 158 gastric ulcer bleeders with different durations of pantoparazole infusion compared to saline infusion.

Outcome (gastric ulcer bleeders)	Saline	Pantoprazole all		Pantoprazole 0–4 hrs		Pantoprazole >4 hrs		Pantoprazole >6 hrs	
	(*n* = 46)	(*n* = 112)	*P* value	(*n* = 65)	*P* value	(*n* = 47)	*P* value	(*n* = 40)	*P* value
	Number of pts. (%)			Number of pts. (%)		Number of pts. (%)		Number of pts. (%)	
Endoscopic signs of bleeding from duodenal ulcers									
Active bleeding	9 (19.5%)	15 (13.4%)	0.32	13 (20.0%)	0.95	2 (4.3%)	**0.02**	2 (5.0%)	**0.04**
Nonbleeding visible vessel	11 (23.9%)	22 (19.6%)	0.55	12 (18.5%)	0.48	10 (21.3%)	0.76	10 (25.0%)	0.91
Clot	5 (10.9%)	28 (25.0%)	**0.04**	20 (30.8%)	**0.01**	8 (17.0%)	0.39	6 (15.0%)	0.57
Pigmented spot and clean base	21 (45.6%)	47 (41.9%)	0.67	20 (30.8%)	0.11	27 (57.4%)	0.26	22 (55.0%)	0.39
Urgent endoscopy	3 (6.5%)	8 (7.1%)	0.82	3 (4.6%)	0.54	5 (10.6%)	0.75	3 (7.5%)	0.65
Recurrent bleeding	4 (8.7%)	18 (16.1%)	0.22	10 (15.4%)	0.29	8 (17.0%)	0.23	7 (17.5%)	0.22
Emergency surgery	2 (4.3%)	6 (5.3%)	0.79	5 (7.7%)	0.48	1 (2.1%)	0.54	1 (2.5%)	0.64
Mortality	1 (2.2%)	8 (7.1%)	0.22	3 (4.6%)	0.49	5 (10.6%)	0.09	4 (10.0%)	0.12

*P* values were calculated for comparison between saline infusion group and pantoprazole infusion group patients with different durations of pantoprazole administration.
